# Cardiac fibroblasts: answering the call

**DOI:** 10.1152/ajpheart.00478.2024

**Published:** 2024-08-02

**Authors:** Petra Kleinbongard, Samuel E. Senyo, Merry L. Lindsey, Alexandra M. Garvin, Jeremy A. Simpson, Lisandra E. de Castro Braz

**Affiliations:** ^1^Institute for Pathophysiology, West German Heart and Vascular Center, University of Essen Medical School, University of Duisburg-Essen, Essen, Germany; ^2^Department of Biomedical Engineering, Case Western Reserve University, Cleveland, Ohio, United States; ^3^School of Graduate Studies, Meharry Medical College, Nashville, Tennessee, United States; ^4^Veterans Affairs Medical Center, Nashville, Tennessee, United States; ^5^Department of Physiology, Brody School of Medicine, East Carolina University, Greenville, North Carolina, United States; ^6^Department of Human Health and Nutritional Sciences, University of Guelph, Guelph, Ontario, Canada; ^7^IMPART Investigator Team Canada, Guelph, Ontario, Canada

**Keywords:** fibrosis, ischemia, myocardium, physiology, pressure overload

## Abstract

Cardiac fibroblasts play a pivotal role in maintaining heart homeostasis by depositing extracellular matrix (ECM) to provide structural support for the myocardium, vasculature, and neuronal network and by contributing to essential physiological processes. In response to injury such as myocardial infarction or pressure overload, fibroblasts become activated, leading to increased ECM production that can ultimately drive left ventricular remodeling and progress to heart failure. Recently, the *American Journal of Physiology-Heart and Circulatory Physiology* issued a call for papers on cardiac fibroblasts that yielded articles with topics spanning fibroblast physiology, technical considerations, signaling pathways, and interactions with other cell types. This mini-review summarizes those articles and places the new findings in the context of what is currently known for cardiac fibroblasts and what future directions remain.

## INTRODUCTION

Cardiac fibroblasts are the primary cell type responsible for the deposition of extracellular matrix (ECM) in the heart, providing support to the contracting myocardium, vasculature, and neuronal network while also contributing to a myriad of physiological and pathophysiological signaling processes (1–3). There is a strong intersection between inflammation and fibrosis in the cardiac response to both acute (e.g., myocardial infarction) and chronic (e.g., hypertension) injuries (4, 5), with these interactions driving fibroblast proliferation and activation leading to ventricular remodeling phenotypes. Fibroblasts are critical indirect mediators of both heart failure progression and the development of arrhythmias through the regulation of changes in ECM that result from fibroblast overactivation (6–9). Cardiac fibroblasts produce ECM that provides structural, mechanical, biochemical, and electrical support to the myocardium, exhibiting remarkable cell plasticity. In response to pathology, fibroblasts undergo a continuum of polarization phenotypes primarily determined by environment and influenced by factors that include age, sex, and genetic regulation (10, 11).

The editorial team of *American Journal of Physiology-Heart and Circulatory Physiology* recently invited submissions related to Cardiac Fibroblasts. We asked for articles describing intramyocardial signaling, cross talk with other cell types, and functionality under physiological and pathophysiological conditions. This Call for Papers was spearheaded by Dr. Petra Kleinbongard, Dr. Samuel Senyo, Dr. Merry Lindsey, Dr. Alexandra (Bobbie) Garvin, Dr. Jeremy Simpson, and Dr. Lisandra de Castro Braz. The call resulted in 12 articles published, which have garnered ∼7,000 downloads and dozens of citations to date.

**Figure 1.**
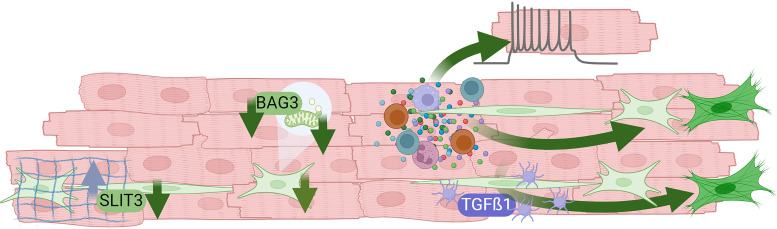
Summary of the contributions of this call to our understanding of the role of the cardiac fibroblast, highlighting Slit homolog 3 (SLIT3) protein, the co-chaperone Bcl2-associated athanogene 3 (BAG3), and transforming growth factor-β1 (TGF-β1) actions. The image was created with a licensed version of BioRender.com.

The purpose of this mini-review is to summarize the articles published in the call, which fall into one of four themes: *1*) reviews of fibroblast physiology; *2*) technical considerations for the use, culturing, and imaging of cardiac fibroblasts; *3*) new signaling information to elucidate how fibroblasts regulate cardiac physiology; and *4*) evidence for cardiac fibroblast interactions with other cell types, including inflammatory cells and platelets (Fig. 1).

## REVIEWS OF FIBROBLAST PHYSIOLOGY

Cardiac fibroblasts maintain both structural and functional homeostasis of the cardiac ECM in a healthy heart and oversee ECM deposition during wound healing responses to injury or disease. Cardiac fibrosis is defined as excessive ECM accumulation, is a common characteristic of numerous cardiovascular diseases including hypertension and myocardial infarction (MI), and often precedes the transition to cardiac dysfunction and heart failure (12–15). Therefore, understanding the physiology of cardiac fibroblasts is crucial for the study of cardiovascular disease onset and progression. Fibroblasts maintain and regulate ECM by facilitating cell-cell communication through ERK, JNK, and p38 signaling pathways stimulated by growth factors, cytokines, and microRNAs (miRNAs) (16–18). Fibroblast signaling includes autocrine and paracrine signaling by secretion of growth factors and cytokines, mechanical sensing, and electrical coupling (19, 20).

Cardiac fibroblasts are key cells in pressure-overload cardiac remodeling that occurs as a result of hypertension, aortic stenosis, or aortic valve stenosis. Although a number of antihypertensive drugs are used clinically and some slow ECM deposition, none was designed to target fibroblasts directly and highlights how different signaling pathways can indirectly regulate fibroblast activation and differentiation. Chalise and Hale review our current knowledge base regarding the diverse populations and activation mechanisms of fibroblasts. In response to hypertension, fibroblasts differentiate into myofibroblasts to yield a fibrogenic phenotype that drives excessive and aberrant ECM accumulation leading to ventricle stiffness, dysfunction, and eventual heart failure (8). The article also examines the antifibrotic effects of antihypertensive therapy, including the persistence of therapy effect even after discontinuation that indicates long-lasting change in cardiac fibroblast phenotype and function (8). As we better understand how the various fibroblast phenotypes are regulated, we can develop targeted antifibrotic therapies that may provide better long-term outcomes for patients with hypertension-induced heart failure. Such antifibrotic therapies would not replace the antihypertensive drugs needed to treat the root pathological cause but rather would serve as an additional measure to preserve the myocardium from excessive ECM accumulation.

The heart is composed of four chambers, and there is a significant regional heterogeneity among the chambers, including structural, functional, and embryonic origin. Much of the research to date has largely targeted the left ventricle for evaluation. The interesting review article by McNair et al. (21) discusses the differences between the right ventricle and left ventricle in terms of fibrosis generation and ECM regulation. They highlight the importance of understanding right ventricle-specific mechanisms of fibrosis, which have often been overlooked in favor of research focused on the left side of the heart. The review emphasizes that fibroblasts in the two ventricles originate from distinct sources (i.e., the right ventricle derives from epicardium and the left ventricle derives from endocardium and epicardium, have side-specific gene signatures, and require distinct approaches to study and treat) (22).

Aberrant and excessive fibrosis is common to all cardiovascular disease, leading to cardiac stiffening. When, how, and for how long cardiac fibroblasts are (and remain) activated depends on the stimuli, chamber (i.e., tissue specific), and microenvironment. The field continues to explore the distinct responses between the ventricles to stress, and challenges remain in understanding how to characterize the complex of fibroblast heterogeneity, which would then allow us to precisely regulate specific subtypes of cells within this diverse cell population.

## TECHNICAL CONSIDERATIONS FOR THE USE, CULTURING, AND IMAGING OF CARDIAC FIBROBLASTS

Cardiac fibroblasts are highly sensitive to mechanical and circulating input making in vitro evaluation of responsiveness to stimuli and phenotypic shifts difficult due to the nonphysiological environment. Thus, much discussion has been made over the years regarding how traditional tissue culture (i.e., tissue culture plastic and growth serum) directly influences the cells and considerations for more accurate conditions for cardiac fibroblasts, including the benefit of using an ex vivo experimental design (1, 11, 15, 23–28). Felisbino and team (29) investigated the impact of substrate stiffness on phenotypic changes of primary ventricular cardiac fibroblasts isolated and cultured from 14-wk-old female rats at varying passage numbers. The authors found that culturing cardiac fibroblasts on low-stiffness hydrogels could prevent an activated state demarcated by the expression of α-smooth muscle actin (α-SMA), while also unexpectedly promoting premature senescence of the cells as indicated by enhanced expression of senescence-associated β-galactosidase, *p16*, and *p21* and increased nuclear factor- kβ (NF-kβ)-mediated inflammatory signaling. The article highlights the importance of considering experimental conditions, including substrate stiffness, when designing experiments in cultured primary cardiac fibroblasts to avoid confounding phenotypic changes that could impact data interpretation.

Following up on methodological considerations for the culture of primary adult cardiac fibroblasts to maintain physiologically relevant conditions, Garvin and Katwa (23) highlight that traditional culture approaches using tissue culture plastic and nutrient-rich media can induce fibroblast activation and alter their subpopulation composition, complicating the interpretation of in vitro results compared with in vivo conditions. The article emphasizes the need for study-specific optimization and validation of culture conditions to ensure the relevance of cardiac fibroblast experiments. Furthermore, ex vivo investigations of cardiac fibroblasts stimulated in vivo and examined in vitro should ideally be complemented by in vivo measurements of fibrosis and fibroblast activation markers to validate mechanism and establish function.

Chen et al. (30) summarize the use of cardiovascular magnetic resonance imaging (MRI) techniques for assessing cardiac fibrosis in mouse models of heart disease. They provide a comprehensive overview of various MRI sequences, parameters, contrast agent delivery methods, and postprocessing strategies used to visualize and quantify different types of cardiac fibrosis, including local and diffuse interstitial fibrosis. The article highlights the potential of MRI-derived biomarkers, such as T1 mapping and extracellular volume fraction, as noninvasive tools for monitoring disease progression and evaluating the efficacy of antifibrotic therapies in preclinical studies.

The takeaway from this collection of articles is that technical considerations for experimental design are important for the interpretation of results. Improved in vivo assessments of fibrosis in addition to single-cell approaches on freshly isolated cardiac fibroblasts are valuable avenues for studies looking to avoid confounding variables of tissue culture. The study of isolated cardiac fibroblasts ex vivo provides meaningful mechanistic insight to molecular signaling responses and should be performed using conditions relevant to the disease model.

## NEW SIGNALING INFORMATION TO ELUCIDATE HOW FIBROBLASTS REGULATE CARDIAC PHYSIOLOGY

A number of miRNAs regulate signaling pathways involved in cardiac fibrosis, and exercise training potentially modulates miRNA signaling to attenuate cardiac fibrosis (31). Specific miRNAs (e.g., miR1, miR29, and miR133) are dysregulated during fibroblast differentiation, proliferation, and activation to generate pathological cardiac remodeling. Improta-Caria et al. (31) reviewed how exercise training can be beneficial to attenuate cardiac fibrosis by modulating the expression of these miRNAs and their associated signaling pathways. This exciting work represents a nonpharmacological strategy to combat aberrant ECM remodeling.

Slit homolog 3 (SLIT3) protein regulates blood vessel development and angiogenesis (32). Gong and Si (33) explored SLIT3 for its potential to serve as a therapeutic target for antifibrotic treatments. SLIT3 mutations in mice lead to abnormalities in collagen-rich connective tissues, suggesting SLIT3 plays a critical role in modulating fibrillar collagen synthesis. The authors found that SLIT3 deficiency mitigated pressure overload-induced cardiac fibrosis and hypertrophy and promoting long-term left ventricle function and animal survival, highlighting SLIT3-mediated fibroblast signaling as a promising target for treating fibrosis. Furthermore, this work highlights how excessive fibrosis directly impacts myocardial remodeling and cardiac function, which ultimately influences survival rates (34).

The co-chaperone Bcl2-associated athanogene 3 (BAG3) is a central node in controlling protein quality in the myocardium. In humans and animal models, reduced BAG3 is linked to cardiac dysfunction and dilated cardiomyopathy (35, 36). Although previous studies focused on BAG3 in cardiomyocytes, the cardiac fibroblasts are critical drivers of pathological remodeling. The Kirk laboratory reported that BAG3 localizes to the mitochondria of primary cardiac fibroblasts and regulates mitophagy (37). Knockdown of BAG3 impairs mitophagy, leading to increased fibroblast activation and differentiation into myofibroblasts, which could explain cardiac fibrosis and remodeling under pathological conditions.

Clinical therapies that specifically target excessive fibrosis are currently lacking. Collectively, the aforementioned manuscripts described recent discoveries that may lead to potential new antifibrotic targets. These included the modulatory effects of exercise in miRNA-dependent reduction of cardiac fibrosis, targeting SLIT3 inhibition to limit collagen synthesis, and regulation of BAG3 levels to maintain fibroblast quiescence. The combined work highlights the need for greater focus on understanding the molecular pathways that regulate fibroblasts and myofibroblasts. The coming years will be exciting as we begin to see the translation of effective therapies to the clinic that specifically target fibrosis.

## EVIDENCE FOR FIBROBLAST INTERACTIONS WITH OTHER CELL TYPES, INCLUDING INFLAMMATORY CELLS AND PLATELETS

Cardiac fibroblasts interact with a number of other cell types in the heart, including cardiomyocytes, endothelial cells, and immune cells (38–40). This cell-cell cross talk is bidirectional, meaning that other cells interact with cardiac fibroblasts to regulate fibrosis. One of the lesser-studied cells to regulate fibroblasts is platelets, small, anucleated blood cells that normally contribute to the formation of a blood clot. Dufeys et al. (41) discuss the role of platelets in cardiac fibrosis, highlighting that early recruitment to the injured myocardium and their ability to secrete factors promotes fibroblast activation and differentiation into myofibroblasts. Platelets are a major source of transforming growth factor-β1 (TGF-β1), a potent profibrotic factor, and other molecules like platelet-derived growth factor, fibroblast growth factor, and a number of chemokines (e.g., interleukin-8, monocyte chemotactic protein-1, and C-C chemokine CCL5) that contribute to fibroblast activation in the heart following MI or pressure overload-induced heart failure (41).

Lymphocytes, particularly T cells and B cells, are primary regulators of cardiac fibroblasts and scar formation following an MI (42). The DeLeon-Pennell laboratory highlights how cross talk between lymphocytes and fibroblasts is bidirectional, with fibroblasts able to activate lymphocytes and lymphocytes able to modulate fibroblast function and ECM synthesis. They emphasize the critical importance of understanding lymphocyte-fibroblast interactions for improving treatment of cardiac fibrosis and heart failure after MI. This concept is further reviewed by the team led by Dr. Pilar Alcaide (7), who summarize the complex cross talk between fibroblasts and immune cells, and how their interplay contributes to cardiac inflammation and fibrosis. Key points of their review include *1*) fibroblasts and immune cells can adopt functions from each other, with fibroblasts acquiring immune-like properties such as cytokine/chemokine secretion and antigen presentation; *2*) fibroblast-immune cell communication is central to cardiac remodeling and the development of heart failure, and *3*) Targeting the interphase of fibroblast and immune cell physiology may provide novel therapeutic approaches to manage inflammation and fibrosis.

The Kohl laboratory provides an overview of the current understanding of nonmyocyte cells, particularly cardiac fibroblasts and macrophages, in cardiac electrical activity and excitation-conduction (43). Although cardiomyocytes were traditionally viewed as the sole contributors to cardiac action potential generation and propagation, recent studies show nonmyocyte cells can also form electrical connections and modulate cardiac electrophysiology. Their review discusses the origins, functions, and basic electrophysiological properties of these nonmyocyte cardiac cell types, emphasizing the importance of considering their contributions to cardiac excitation and arrhythmogenesis.

Together, these papers highlight how nonmyocytes (i.e., fibroblasts, immune cells, and platelets) play a largely unappreciated role in regulating cardiac fibrosis and electrophysiological properties in health and disease. This work showcases the complex interplay of cellular and molecular mechanisms as we move away from seeing the heart as mainly a cardiomyocyte pump and begin to tie in others resident and infiltrating cells (14). The main challenges moving forward lie in untangling these cellular networks to develop new ways to prevent and potentially regress pathological ECM remodeling (44).

The articles in this special call collectively underscore the complex role of cardiac fibroblasts in regulating ECM. Excessive ECM accumulation leads to stiffening of the myocardium with development of dysfunction, which increases the mortality rate. Despite the clinical appreciation of the negative contributions of fibrosis, we lack effective therapies that specifically target cardiac fibrosis. Potential targets, such as miRNAs, SLIT3, BAG3, and platelets have emerged in the past few years and are promising new avenues. Challenges remain in understanding the intricate molecular and cellular cross talk between all cardiac cell types, and how this differs between the cardiac chambers or are influenced by sex and age. The heterogenicity of the cardiac fibroblast population is an evolving field and there is a growing need for better standardized methods and markers that can be applied across all models of heart failure (11, 15). Furthermore, development of diagnostic tools and methods to noninvasively and accurately image fibrosis, not just to quantify but also to assess the quality and degree of stiffness is essential for monitoring disease progression and therapeutic efficacy. The next few years will be exciting as we see more preclinical studies identifying new drugs that will pave the way for clinical trials. Combined, these articles provide the latest information we have on cardiac fibroblasts and serve as excellent resources for those examining this cell type.

To describe the complex functions and plasticity of cardiac fibroblasts, we quote the British biochemist Prof. Michael Denton: “To grasp the reality of life as it has been revealed by molecular biology, we must magnify a cell a thousand million times…. What we would then see would be an object of unparalleled complexity and adaptive design…we would find ourselves in a world of supreme technology and bewildering complexity” (45). This is an exciting time to be involved in the field of cardiac fibroblast physiology.

## GRANTS

The study was funded by the National Institutes of Health (NIH) Grants NS121374 (to S.E.S.), GM151274 (to M.L.L.), and HL152297 (to L.E.d.C.B.); Biomedical Laboratory Research and Development Service of Veterans Affairs Office of Research and Development Grant 5I01BX000505 (to M.L.L.). J.A.S. was funded by the Natural Sciences and Engineering Research Council of Canada (NSERC), the Canadian Institutes of Health Research (CIHR), and the Heart and Stroke Foundation of Canada. We also acknowledge funding from NIH for conferences that stimulated discussion leading to the call for papers that is the subject of this paper, including R13HL168861 and R13HL165779.

## DISCLAIMERS

The content is solely the responsibility of the authors and does not necessarily represent the official views of any of the funding agencies or the American Physiological Society. All authors have reviewed and approved the article.

## DISCLOSURES

M. L. Lindsey and P. Kleinbongard are editors of the *American Journal of Physiology-Heart and Circulatory Physiology* and were not involved and did not have access to information regarding the peer-review process or final disposition of this article. An alternate editor oversaw the peer-review and decision-making process for this article. None of the other authors has any conflicts of interest, financial or otherwise, to disclose.

## AUTHOR CONTRIBUTIONS

P.K., S.E.S., A.M.G., J.A.S., and L.E.d.C.B. conceived and designed research; P.K. prepared figures; L.E.d.C.B. drafted manuscript; P.K., S.E.S., A.M.G., J.A.S., and L.E.d.C.B. edited and revised manuscript; P.K., S.E.S., A.M.G., J.A.S., and L.E.d.C.B. approved final version of manuscript.
